# A Treatise on Artificial Teeth and Palates, and Their Importance and Cure of Dyspepsia, &c.

**Published:** 1846-06

**Authors:** 


*A Treatise on Artificial Teeth and Palates, and their importance and cure of Dyspepsia, &c.* By Horatio Pass, Surgeon Dentist, Lecturer on Anatomy and Physiology of the Teeth; 16mo, pp. 98. London, Churchhill, 1846.

The above work is a new compilation of a variety of sentences by able authors, *tacked* together and intended, we presume, to explain, more elaborately, the author's diurnal advertisement, in the English press, on his recently discovered *secret* odontalgic remedies. R.

